# Hydroxylated Dimeric Naphthoquinones Increase the Generation of Reactive Oxygen Species, Induce Apoptosis of Acute Myeloid Leukemia Cells and Are Not Substrates of the Multidrug Resistance Proteins ABCB1 and ABCG2

**DOI:** 10.3390/ph9010004

**Published:** 2016-01-19

**Authors:** Rena G. Lapidus, Brandon A. Carter-Cooper, Mariola Sadowska, Eun Yong Choi, Omasiri Wonodi, Nidal Muvarak, Karthika Natarajan, Lakshmi S. Pidugu, Anil Jaiswal, Eric A. Toth, Feyruz V. Rassool, Arash Etemadi, Edward A. Sausville, Maria R. Baer, Ashkan Emadi

**Affiliations:** 1 Marlene and Stewart Greenebaum Cancer Center, School of Medicine, University of Maryland, Baltimore, MD 21201, USA; RLapidus@som.umaryland.edu (R.G.L.); bcooper@som.umaryland.edu (B.A.C.-C.); mariolas349@gmail.com (M.S.); echoi@som.umaryland.edu (E.Y.C.); omasiri1@hotmail.com (O.W.); nmuvarak@som.umaryland.edu (N.M.); Karthika.natarajan001@gmail.com (K.N.); ajaiswal@som.umaryland.edu (A.J.); frassool@som.umaryland.edu (F.V.R.); esausville@umm.edu (E.A.S.) mbaer@umm.edu (M.R.B.); 2 Department of Medicine, School of Medicine, University of Maryland, Baltimore, MD 21201, USA; 3 Center for Biomolecular Therapeutics, School of Medicine, University of Maryland, Baltimore, MD 20850, USA; lpidugu@som.umaryland.edu (L.S.P.); etoth@som.umaryland.edu (E.A.T.); 4 Department of Pharmacology, School of Medicine, University of Maryland, Baltimore, MD 21201, USA; 5 Department of Biochemistry and Molecular Biology, School of Medicine, University of Maryland, Baltimore, MD 21201, USA; 6 Department of Radiation Oncology, School of Medicine, University of Maryland, Baltimore, MD 21201, USA; 7 Division of Cancer Epidemiology and Genetics, National Cancer Institute, National Institutes of Health, Bethesda, MD 20892, USA; arash.etemadi@nih.gov

**Keywords:** dimeric naphthoquinone, acute myeloid leukemia (AML), oxidative stress, reactive oxygen species (ROS)

## Abstract

Selective targeting of the oxidative state, which is a tightly balanced fundamental cellular property, is an attractive strategy for developing novel anti-leukemic chemotherapeutics with potential applications in the treatment of acute myeloid leukemia (AML), a molecularly heterogeneous disease. Dimeric naphthoquinones (BiQs) with the ability to undergo redox cycling and to generate reactive oxygen species (ROS) in cancer cells are a novel class of compounds with unique characteristics that make them excellent candidates to be tested against AML cells. We evaluated the effect of two BiQ analogues and one monomeric naphthoquinone in AML cell lines and primary cells from patients. All compounds possess one halogen and one hydroxyl group on the quinone cores. Dimeric, but not monomeric, naphthoquinones demonstrated significant anti-AML activity in the cell lines and primary cells from patients with favorable therapeutic index compared to normal hematopoietic cells. BiQ-1 effectively inhibited clonogenicity and induced apoptosis as measured by Western blotting and Annexin V staining and mitochondrial membrane depolarization by flow cytometry. BiQ-1 significantly enhances intracellular ROS levels in AML cells and upregulates expression of key anti-oxidant protein, Nrf2. Notably, systemic exposure to BiQ-1 was well tolerated in mice. In conclusion, we propose that BiQ-induced therapeutic augmentation of ROS in AML cells with dysregulation of antioxidants kill leukemic cells while normal cells remain relatively intact. Further studies are warranted to better understand this class of potential chemotherapeutics.

## 1. Introduction

The combination of cytarabine and an anthracycline as the mainstay of treatment for acute myeloid leukemia (AML) has not changed significantly for the last forty years and has resulted in an approximately 30%–40% 5-year survival rate in patients younger than 65 years of age [1]. The problem of inadequate treatment options for AML is exacerbated by an upsurge in the incidence of AML to approximately 20,830 new cases in the US in 2015 [2,3]. Thus, there is an urgent need for development of new therapeutic strategies and agents for AML treatment. Existing mutational targeted inhibitors have not proved to be curative or strikingly effective clinically, perhaps because of the multi-hit nature of leukemogenesis [4]. AML cells in particular those with fms-like tyrosine kinase 3 internal tandem duplication (FLT3/ITD) mutations generate increased ROS [5,6]; hence, there may be a threshold level of extra oxidative stress that these cells can tolerate. Therefore, perturbing the cellular oxidative state appears to be selective for eradication of AML cells due to its differential effects in primary AML cells and normal hematopoietic cells [7,8,9]. Moreover, the cellular oxidative state is a universal target in AML cells irrespective of the biologic heterogeneity of AML [8].

A class of compounds that has shown great promise in targeting the cellular oxidative state are the quinones, which are broadly distributed in Nature [10]. Historically, several synthetic and natural quinones, including anthracyclines, mitoxantrone, and mitomycin-C, have demonstrated significant antineoplastic activity, resulting in broad usage in many hematologic and solid neoplasms [11,12]. A particularly promising quinone family is the naphthoquinones, with antibacterial, antifungal, antiviral, and anti-neoplastic derivatives [13]. In an effort to regioselectively synthesize conocurvone, a naturally occurring trimeric naphthoquinone with potent anti-HIV activity [14,15], we synthesized a new set of dimeric naphthoquinones or bi-naphthoquinones (BiQs) [16,17,18]. We determined structure-activity relationships (SARs) of 12 BiQ analogs on the growth of prostate and breast cancer cell lines and found that these compounds selectively exert their anticancer activity via perturbation of cellular oxidative states [19,20].

To better understand the cellular mechanisms involved in the cytotoxicity of BiQs, we performed a chemical genetic screen in yeast and found that the yeast oxidoreductase Nde1 was the major target of BiQs [21]. The human homologue of Nde1 is NAD(P)H quinone oxidoreductase 1 (E.C. 1.6.99.2, NQO1, also known as DT-diaphorase and NAD(P)H dehydrogenase, quinone 1). We have recently described an extensive binding interface between a bromohydroxy BiQ and the isoalloxazine ring of the flavin adenine dinucleotide (FAD) cofactor of NQO1, in addition to interactions with protein side chains in the active site (in press). Here, we report the cytotoxic effect of two halohydroxy dimeric naphthoquinones, BiQ-1 and BiQ-2, on human AML cell lines and primary cells from patients. We also describe the oxidation/reduction (redox) consequences of exposure to BiQs in AML cells, and preliminary tolerability studies in mice.

## 2. Results

### 2.1. Dimeric Naphthoquinones Decreased Viable Numbers of AML Cell Line and Primary Cells with Favorable Therapeutic Index in Relation to Normal Hematopoietic Cells

**Figure 1:**
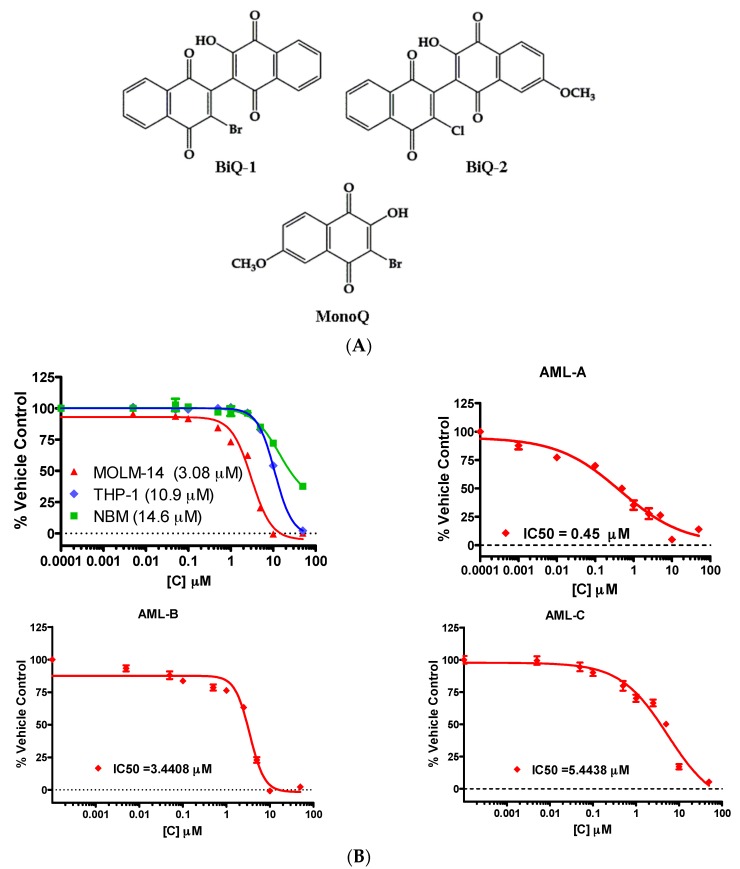

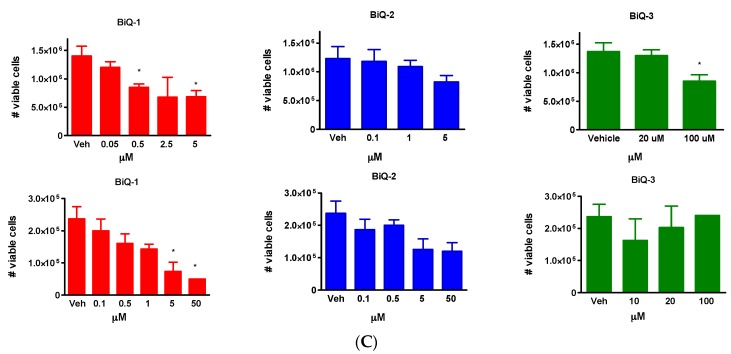
(**A**) Structures of BiQ-1, BiQ-2 and MonoQ; (**B**) MOLM-14 and THP-1 cells as well as AML-A, AML-B, AML-C and normal bone marrow cells from a healthy donor were exposed to a range of BiQ-1 concentrations. Cells were cultured in the presence of BiQ-1 for 72 h (48 h for primary cells) and Alamar Blue reagent was then added. The representative growth inhibition curves for each cell are shown in this Figure. Mean ± standard deviation IC_50_ values are shown in Table 1; (**C**) BiQ-1 inhibits AML cell growth regardless of FLT3 mutation status. Cell survival after BiQ exposure was determined via trypan blue exclusion. MOLM-14 and THP-1 were treated with BiQ-1, BiQ-2 or MonoQ at serial concentrations for 72 h. Both BiQ-1 and BiQ-2 induced a concentration-dependent reduction in cell survival, while only a slight, non-significant, decrease in viable cells was observed even with 100 µM MonoQ in the MOLM-14 cells (* *p* < 0.05). Only BiQ-1 produced a statistically significant decrease in cell numbers at its IC_50_ concentration in the trypan blue exclusion assay (* *p* < 0.05).

We first tested the ability of two dimeric halohydroxy naphthoquinones, BiQ-1 and BiQ-2 (Figure 1A) [16], to decrease proliferation of two AML cell lines and primary AML cells from three patients. To test the necessity of the dimeric moiety in the naphthoquinone structure, we also tested the cytotoxicity of one monomeric halohydroxy naphthoquinone, MonoQ, against AML cells. We observed a concentration-dependent decrease in metabolic activity of MOLM-14 and THP-1 cells with exposure to BiQ-1 (representative data in Figure 1B; Table 1) and BiQ-2 (Table 1) for up to 72 h. These two compounds were also cytotoxic toward the primary cells, AML-A, AML-B and AML-C (the first two with FLT3-WT and the third with FLT3-ITD) (representative data in Figure 1B; Table 1). MonoQ did not show any cytotoxic effect, underscoring the requirement for the presence of two attached naphthoquinones (*i.e.*, dimer) for anti-leukemic activity. Moreover, the IC_50_s of BiQs for normal bone marrow cells were approximately three times higher than those for most of the AML cells, suggesting a favorable therapeutic index of these agents (Figure 1B; Table 1).

After 72 h exposure to BiQ-1 or BiQ-2, there were significantly fewer viable MOLM-14 and THP-1 cells, compared with vehicle control (Figure 1C). BiQ-1 was more cytotoxic than BiQ-2 toward both AML cell lines (Figure 1C). MonoQ had no or very little effect on the survival of AML cells even at 100 µM concentration (Figure 1C). Due to the inherently fragile state of the primary AML cells and very poor viability after thawing, cell survival could not be tested in the primary AML cells. Because of its superior potency, BiQ-1 was selected for testing in the remaining experiments and for elucidation of mechanisms of action.

**Table 1:** IC_50_ values of BiQ-1, BiQ-2, and MonoQ in leukemia cell lines and primary AML cells isolated from patients, and normal bone marrow cells.

Cell Line	BiQ-1 (µM)	BiQ-2 (µM)	MonoQ (µM)
MOLM-14 (complex karyotype, FLT3-ITD)	3.1 ± 0.2 **	4.5 ± 1.9 **	>100 (NE)
THP-1 (complex karyotype, FLT3-WT)	8.5 ± 4.2 *	8.6 ± 4.3	>100 (NE)
AML-A (46,XY; FLT3-WT)	0.36	3	>100 (NE)
AML-B (46,XY; FLT3-WT)	3.3 ± 0.3 *	NT	NT
AML-C (complex karyotype; relapsed post-transplant; FLT3-ITD)	5.1 ± 0.7 *	NT	NT
Normal BM	14.6	14.1	NT

IC_50_ values were calculated as mean ± standard deviation from at least two independent experiments (72 h exposure for cell lines and 48 h exposure for primary cells). If availability of primary blasts was limited, the IC_50_ was obtained from a single experiment. BM = bone marrow; FLT3: Fms-like tyrosine kinase 3; IC_50_: concentration that decreases viable cell numbers by 50%; ITD = internal tandem duplication; NE = Not Effective; NT = Not Tested; WT = wild type. * *p* < 0.05; ** *p* < 0.0001 compared with the value for normal BM.

### 2.2. BiQ-1 Induces Apoptosis of AML Cells

**Figure 2:**
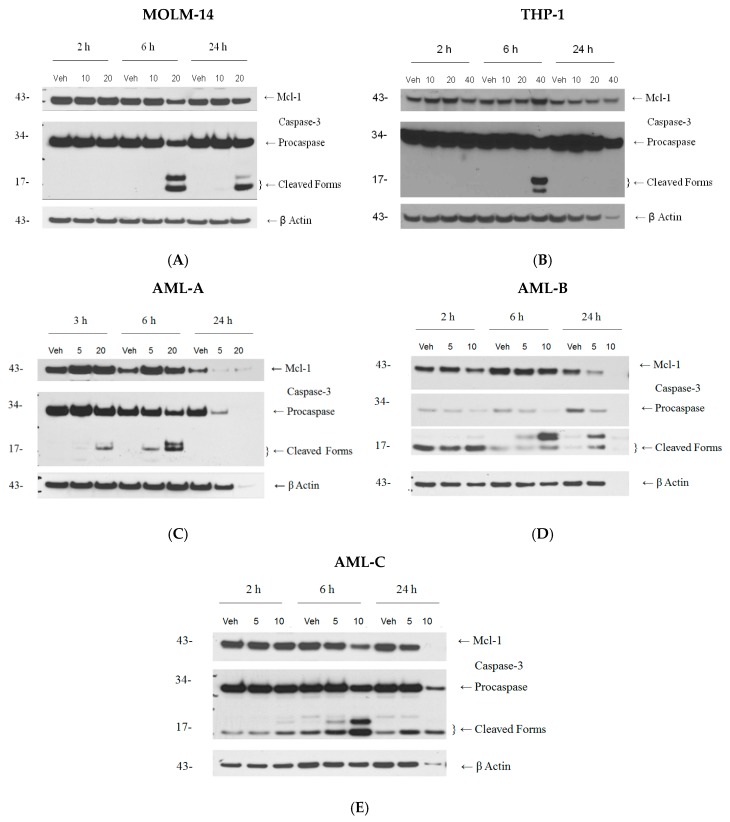
BiQ-1 induced apoptosis of AML cells as measured by Western Blot. (**A**) BiQ-1 treatment of MOLM-14 cells resulted in reduced Mcl-1 expression at 6 and 24 h post-treatment; (**B**) In contrast, at 6 and 24 h post-treatment in THP-1, a concentration-dependent increase in Mcl-1 expression was observed. (**A**,**B**) In MOLM-14 and THP-1, caspase-3 cleavage was observed after 6 h of treatment with 20 µM and 40 µM BiQ-1, respectively. Caspase-3 cleavage was maintained at 24 h in MOLM-14, but not THP-1; (**C**–**E**) In concentrations ranging from 10 µM to 20 µM, BiQ-1 induced caspase-3 cleavage within 6 h in primary AML cells from patients.

We have demonstrated that BiQ-1 decreased AML cell line and primary patient sample viable cell numbers at low micromolar concentrations. To determine whether BiQ-1 was cytotoxic, as opposed to cytostatic, we measured whether cells were dying via apoptotic pathways using three different assays: Western Blot analysis, annexin V staining, and mitochondrial membrane depolarization. By Western Blot analysis, BiQ-1 induced downregulation of Mcl-1 and cleavage of caspase-3, both hallmarks of induction of apoptosis. In MOLM-14 cells, caspase-3 cleavage was observed at 20 µM at 6 and 24 h (Figure 2), while caspase-3 cleavage was seen at higher concentration in THP-1 cells and at lower concentrations in primary cells AML-A, AML-B and AML-C (Figure 2). Primary AML cells AML-B and AML-C demonstrated some levels of caspase-3 cleavage with exposure to vehicle alone, denoting the fragile state of these primary leukemia cells in culture. Hence, to avoid false positive results, we decided to test only MOLM-14 and AML-A cells in the flow cytometric assays evaluating annexin V staining and mitochondrial membrane potential.

**Figure 3:**
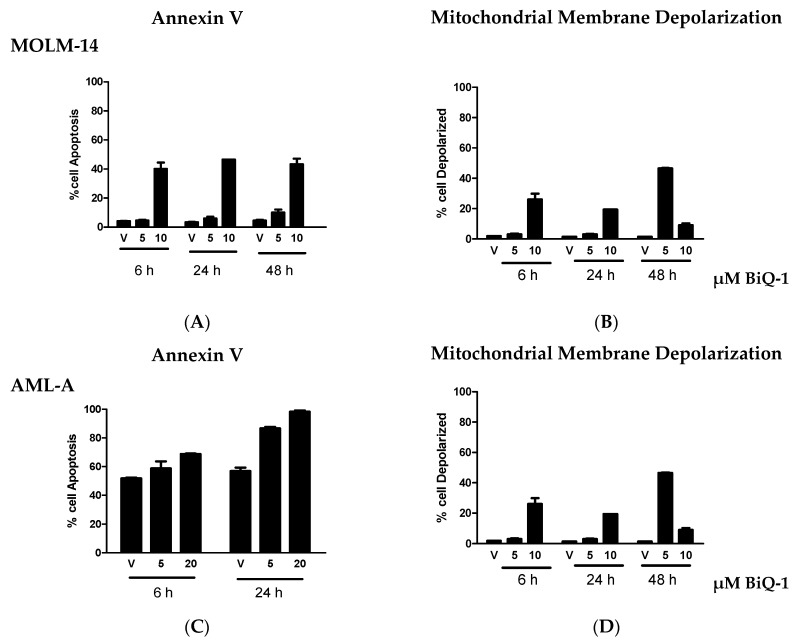
BiQ-1 induced apoptosis and mitochondrial membrane depolarization, as measured by annexin V and MitoPotential-Red. (**A**,**C**) MOLM-14 and (**B**,**D**) AML-A cells were treated with 5–20 µM of BiQ-1 and cells were collected and analyzed by flow cytometry 6, 24 and 48 h after exposure. * *p* < 0.05 compared to vehicle. Representative data is shown.

As measured by annexin V staining, the number of cells undergoing apoptosis increased by 9.7-, 13.7- and 9.6-fold at 6, 24 and 48 h, respectively, in MOLM-14 cells treated with 10 µM BiQ-1, compared to vehicle control (Figure 3A). Note, cells treated with 10 µM BiQ-1 had significantly more annexin V stained cells than Vehicle (*p* < 0.05). In AML-A cells, there was more than 50% apoptosis of cells when treated with vehicle alone and very little enhancement of apoptosis was observed at 6 h, but at 24 h level of apoptosis increased by 50% and 70% in cells treated with 5 and 20 µM BiQ-1, respectively, compared to vehicle control (Figure 3C, bottom left; *p* < 0.05).

To further investigate the mechanism of apoptosis, we used flow cytometry with MitoPotential Red stain to test whether BiQ-1 treatment induced depolarization of the mitochondrial transmembrane potential (ΔΨm), resulting in release of apoptogenic factors. Upon exposure to 5 µM and 10 µM BiQ-1, 1.6-, 1.9- and 32-fold and 13.8-, 13.3- and 6.2-fold more MOLM-14 cells were observed with mitochondrial membrane depolarization at 6, 24 and 48 h, respectively (Figure 3B). In MOLM-14 cells, while only 10 µM BiQ-1 significantly induced mitochondrial membrane depolarization at 6 and 24 h, both 5 and 10 µM significantly enhanced depolarization at 48 h (*p* < 0.05). Additionally, 5 µM and 20 µM BiQ-1 induced 1.6, and 1.9-fold increase in AML-A cells with mitochondrial membrane depolarization at 24 h, respectively (*p* < 0.05) (Figure 3D). Significant induction of mitochondrial membrane depolarization was observed in AML-A cells after 6 h exposure to 20 µM BiQ-1 (*p* < 0.05).

### 2.3. ROS Induction is Evident after BiQ-1 Exposure

**Figure 4:**
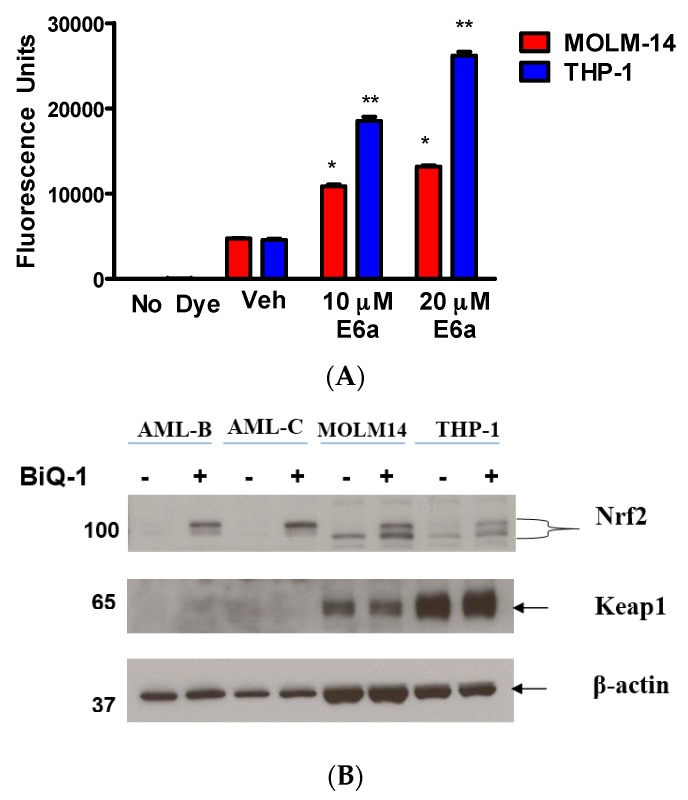
BiQ-1 treatment increased cellular ROS levels in AML cells. (**A**) MOLM-14 and THP-1 cells were loaded with H_2_DCFA dye for 30 min and then exposed to 10 and 20 µM of BiQ-1 for 2 h. Both cell lines displayed a significant increase in ROS (* *p* < 0.00001; ** *p* < 0.00001); (**B**) Nrf2 and Inrf2/Keap1 induction after exposure to BiQ-1. MOLM-14 and THP-1 cell lines were treated with 5 µM BiQ-1 for 2 h.

We and others have shown that naphthoquinones are able to undergo redox cycling inside the cells and generate reactive oxygen species (ROS), including superoxide and peroxide [21,22]. To investigate whether dimeric naphthoquinones increased cellular ROS in AML cells, we measured ROS levels by flow cytometry after exposure of the cells to BiQ-1. Two hours of treatment with BiQ-1 at 10 and 20 µM concentration increased cellular ROS levels 2.3- and 2.7-fold in MOLM-14 cells and 4.1- and 5.7-fold in THP-1 cells, as compared to vehicle-treated cells (Figure 4A). A no dye control in the presence of BiQ-1 was included in the experiment since BiQ-1 has slight auto-fluorescence, but no significant fluorescence was present due to BiQ-1 alone.

To evaluate the cellular response to the increased ROS levels after exposure to BiQ-1, we measured changes in expression of Nrf2 and Keap1, which are the major transcriptional regulators of the expression of antioxidant proteins in response to cellular oxidative stress [23]. Nrf2 was up-regulated in MOLM-14 and THP-1 cells, after 2 h exposure to 5 µM BiQ-1, indicating the induction of oxidative stress by ROS induced by BiQ-1 (Figure 4B).

### 2.4. BiQ-1 Inhibits Clonogenic Growth of AML Cell Lines

**Figure 5:**
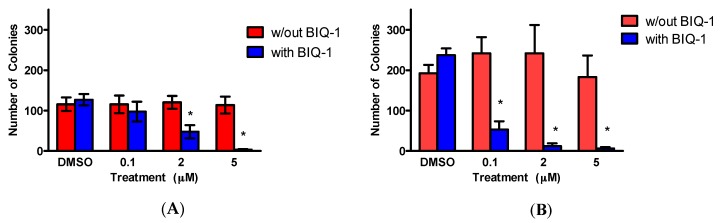
BiQ-1 inhibits clonogenic growth of MOLM-14 (**A**) and THP-1 (**B**) cells. AML cells were treated with BiQ-1 at three concentrations for 24 h and subsequently plated in methylcellulose with or without BiQ-1 at the pre-plating concentrations. Cells grew for 7–12 days prior to termination of the experiment. In the presence of BiQ-1 in the methylcellulose, a significant concentration-dependent reduction in clonogenic growth was observed (* *p* < 0.05) compared to vehicle (DMSO) control. Clonogenic inhibition was not observed when BiQ-1 was not present in the methylcellulose. The data are representative of two experiments.

MOLM-14 and THP-1 cells were exposed to BiQ-1 at three concentrations (0.1, 2, and 5 µM) for 24 h prior to plating in clonogenic assays. Cells were then plated in methylcellulose with and without BiQ-1. BiQ-1 did not inhibit clonogenic growth of cells that were only exposed prior to plating. In contrast, when cells were exposed to BiQ-1 prior to plating and then plated with BiQ-1, the dimeric naphthoquinone significantly inhibited clonogenic growth of both MOLM-14 and THP-1 cells (*p* < 0.05; Figure 5).

### 2.5. BiQ-1 Is Not a Substrate of the ATP Binding Cassette (ABC) Transporters

**Figure 6:**
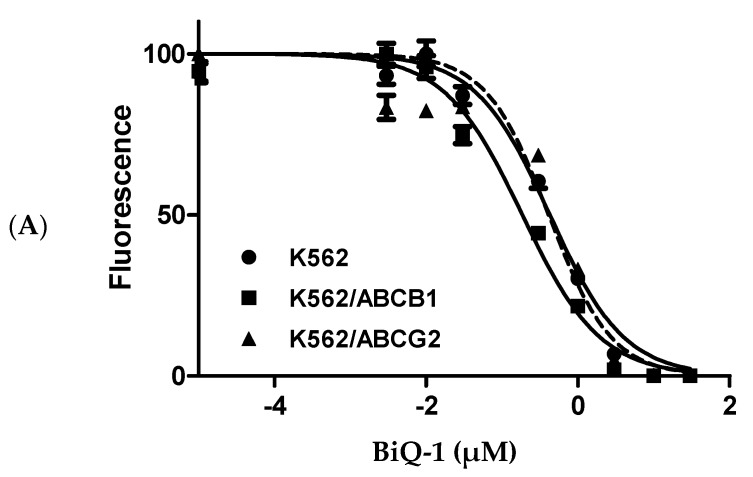

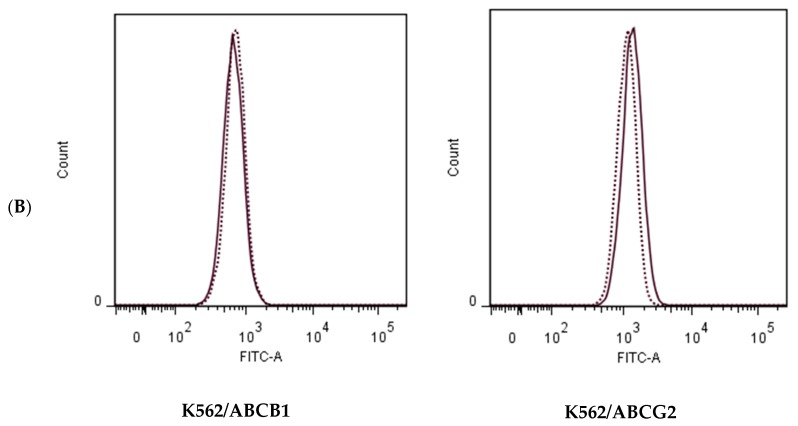
ABCB1 and ABCG2 do not confer resistance to BiQ-1. (**A**) BiQ-1 cytotoxicity in cells overexpressing ABCB1 (K562/ABCB1), ABCG2 (K562/ABCG2) and parental cells (K562) is shown. Viability of BiQ-1-treated cells was evaluated using the WST-1 assay, and IC_50_s were calculated as described in Materials and Methods; (**B**) BiQ-1 uptake was similar in ABCB1- and ABCG2-overexpressing cells in the presence of absence of the ABCB1-specific inhibitor PSC-833 or ABCG2 inhibitor fumitremorgin C.

Multidrug resistance (MDR) has been implicated as a mechanism of chemotherapy failure in AML. Anthracyclines, quinone-based chemotherapeutics that are the backbone of chemotherapy regimens commonly used to treat AML, are substrates of the ATP-binding cassette protein drug efflux pumps ABCB1 (P-glycoprotein) and ABCG2 (breast cancer resistance protein), which are expressed on AML cells and other cancer cells [24]. To test whether dimeric naphthoquinones are substrates for these drug efflux pumps, BiQ-1 was tested in two distinct assays. The cytotoxicity of BiQ-1 against K562 leukemic cells with and without overexpression of ABCB1 or ABCG2 was evaluated. BiQ-1 was equally potent at inhibiting cell proliferation in cells overexpressing ABCB1 and ABCG2 and parental cells (K562—0.42 µM; K562/ABCB1—0.19 µM; K562/ABCG2—0.43 µM) (Figure 6A), demonstrating that BiQ-1 is not a substrate of the two ABC transporters tested. Additionally, there were no significant differences in uptake of BiQ-1, measured by flow cytometry, in cells overexpressing ABCB1 or ABCG2 in the presence and absence of the ABCB1- or ABCG2-specific transport inhibitors PSC-833 or fumitremorgin C (Figure 6B).

### 2.6. BiQ-1 Was Well Tolerated in Female Swiss Webster Mice

**Figure 7:**
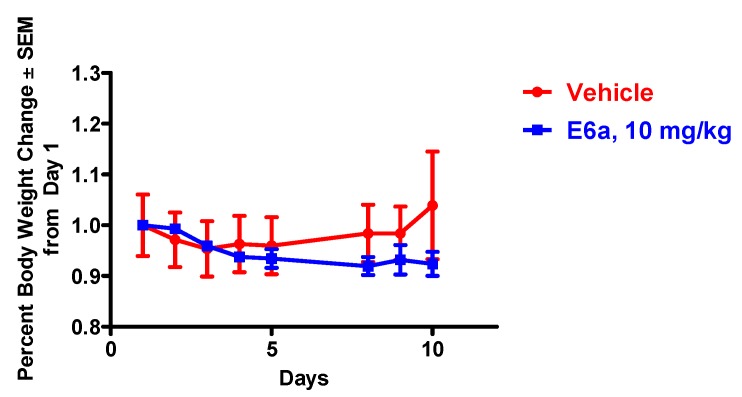
The effect of BiQ-1 in female Swiss Webster mice. BiQ-1 was dosed daily for 5 days, two days off and then continued for 3 more days. Mean body weight loss did not exceed 10%. Three mice were included per group.

In order to evaluate the tolerability and potential toxicities of BiQ-1 *in vivo*, female Swiss Webster mice were treated with 10 and 25 mg/kg BiQ-1 delivered by intraperitoneal (IP) injection on days 1 through 5 and 8 through 10. Mice dosed with 10 mg/kg IP displayed no overt signs of toxicity and lost very little weight during the experiment (Figure 7). The 25 mg/kg dose was toxic; mice became lethargic and stopped ambulating, eating and drinking after two doses. These mice were euthanized due to toxicity. In contrast, mice dosed with 25 mg/kg BiQ-1 subcutaneously on same schedule showed no weight loss and no overt signs of toxicity, except for slight irritation at the injection sites. Thus the toxicity in the mice receiving 25 mg/kg BiQ-1 by IP injection may have been due to peritoneal irritation from the acidic pH of the compound. Complete blood counts (CBC) of mice dosed with 10 mg/kg BiQ-1 showed slight leukocytosis, increased neutrophil count and erythrocytosis in one of two mice compared to vehicle alone (Table 2). Additionally, BiQ-1 up to 10 mg/kg caused no overt renal or hepatic toxicity in mice.

**Table 2:** Complete Blood Count findings in mice treated with BiQ-1.

	Vehicle		BiQ-1 (10 mg/kg)		Normal Range	Units
**ID number of mouse**	**1**	**3**	**2**	**4**		
White Blood Cell	9.46	9.68	4.80	16.73	3.2–12.7	(× 10^3^ cells/µL)
Neutrophil	5.90	7.30	16.40	23.50		%
Lymphocyte	86.80	82.90	61.70	62.10		%
Monocyte	1.80	2.00	3.00	3.10		%
Eosinophil	4.40	5.40	17.70	10.10		%
Basophil	0.20	0.20	0.30	0.20		%
Red Blood Cell	8.11	10.02	7.28	11.07	7.0–10.1	(× 10^3^ cells/µL)
Hemoglobin	13.50	15.90	11.50	17.50	11.8–14.9	mg/dL
Hematocrit	46.10	55.70	40.40	62.20	36.7–46.8	%
Mean corpuscular volume	56.90	55.60	55.50	56.20	42.2–59.2	fL
Platelet	1168	1328	1611	1944	766–1657	(× 10^3^ cells/µL)

## 3. Discussion

Quinones, including naphthoquinones, possess unconjugated electrons and participate in chemical reactions transferring those electrons, which culminate in the generation of highly active oxygen radicals. At low intracellular levels, ROS contribute to normal signaling function. However, when ROS occur in excess, damage to cellular components including proteins, lipids and DNA occurs. Irrespective of their genetic characteristics, leukemia cells with high growth fractions generate an excess of ROS. In turn, leukemia cells have prominent antioxidant machinery to mitigate the effects of excess ROS and maintain cellular oxidative states compatible with cell survival [25,26]. Thus, mechanisms maintaining the oxidative state in leukemia cells may be exploitable targets to selectively eradicate neoplastic cells [7], as compared to normal cells.

The role of quinone analogs in electron transfer reactions varies depending on their structures [27,28]. Through electrochemical studies, we have demonstrated that a dimeric naphthoquinone can undergo four redox steps, in which the cathodic and anodic peak potentials can be tuned, with the ultimate outcome of generating four moles of superoxide radical per mole of naphthoquinone. Our goal is to generate ROS to target cellular metabolic processes in malignant cells, with the possibility of avoiding indiscriminate structural damage.

We hypothesize that AML cells are particularly sensitive to oxidative stress induced by dimeric naphthoquinones because they have higher endogenous levels of ROS and antioxidants than normal cells, and hence lower compensatory reserves for handling additional ROS. In this study, we have shown that the novel halohydroxy dimeric naphthoquinones BiQ-1 and BiQ-2 have low micromolar potency against both FLT3-WT and FLT3-ITD AML cell lines and primary cells from patients with AML. MonoQ, a bromohydroxy mononaphthoquinone, did not show an anti-leukemic effect, indicating the importance of the presence of the dimer, with the potential to undergo four redox steps. BiQ-1 decreased viable AML cells and decreased clonogenic growth of AML cells. Moreover, it induced apoptosis of AML cells. Importantly, it appears that dimeric naphthoquinones have a selective effect on leukemic cells, with a reasonable therapeutic index, as evidenced by the fact that the cytotoxic effect of BiQ-1 on normal hematopoietic cells occurred at approximately three times higher concentrations than those required for killing AML cells, and the fact that BiQ-1 at 10 mg/kg IP injections for eight days in mice was well tolerated and did not significantly affect blood counts or renal or hepatic function. Further studies to evaluate the safety and efficacy of BiQ analogues when administered *in vivo* to inhibit growth of primary human AML cells, as well as pharmacokinetic and pharmacodynamic studies, are planned.

From a mechanistic point of view, as expected, BiQ-1 increased oxidative stress in AML cells by enhancing ROS levels, which resulted in an increased expression of Nrf2, the master regulator of cellular protection against oxidative hazards. Finally, we demonstrated that, unlike anthracyclines, BiQ-1 is not a substrate for the drug efflux proteins ABCB1 or ABCG2.

## 4. Materials and Methods

### 4.1. Chemicals and Reagents

The one step reaction between commercially available 2,3-dibromo-1,4-naphthoquinone and 2,3-dichloro-1,4-naphthoquinone with 2-hydroxynaphthoquinones in presence of cesium carbonate in acetonitrile at room temperature yielded 3-bromo-3′-hydroxy-2,2′-binaphthalenyl-1,4,1′,4′-tetraone (BiQ-1) and 3′-chloro-3-hydroxy-7-methoxy-2,2′-binaphthalenyl-1,4,1′,4′-tetraone (BiQ-2), respectively, as yellow powders with 94% yield, as previously described [16]. Monomeric halohydroxy naphthoquinone, 3-bromo-2-hydroxy-6-methoxy-1,4-dihydro-1,4-naphthalenedione (MonoQ), was synthesized as previously described [16]. Structures were confirmed using nuclear magnetic resonance (^1^H-NMR and ^13^C-NMR) as well as low- and high-resolution mass spectrometry using fast atom bombardment (FAB) or electron impact (EI), as reported in Emadi *et al.* [16] All drugs were dissolved in DMSO.

### 4.2. Cell Lines and Patient Cells

MOLM-14 cells were the kind gift of Dr. Mark Levis, Johns Hopkins University. MOLM-14 cells carry the FLT3-ITD mutation. THP-1 cells were purchased from American Type Culture Collection (ATCC, Manassas, VA, USA). THP-1 cells have wild type FLT3 (FLT3-WT). Primary human leukemia cells AML-A, AML-B and AML-C were obtained through an institutional (Institutional Review Board (IRB) approved) tissue procurement protocol at the University of Maryland Greenebaum Cancer Center. Briefly, whole blood was received in sodium ethylenediaminetetraacetate (EDTA) tubes, and diluted 1:1 with phosphate-buffered saline (PBS). Cells were isolated from diluted whole blood by density separation in lymphocyte separation medium (Corning Cellgro, Manassas, VA, USA) spun at 400× *g* for 30 min with no brake. Healthy donor bone marrow was received from the Interstate Companies (Memphis, TN, USA) and peripheral blood mononuclear cells (PBMCs) were isolated in the same manner. Viable cells were counted using trypan blue exclusion. All cell lines and primary cells were cultured at 37 °C in 5% CO_2_ atmosphere in Roswell Park Memorial Institute (RPMI) 1640 medium (Life Technologies, Carlsbad, CA, USA) supplemented with heat-inactivated 10% (*v*/*v*) fetal bovine serum (FBS). Cell lines were grown and maintained according to American Type Culture Collection (ATCC) recommendations.

### 4.3. Cell Proliferation Assay

Cell lines and primary cells were seeded in 96-well plates the afternoon prior to treatment. Approximately 18 h later, BiQ-1, BiQ-2 and MonoQ were semi-serially diluted in dimethyl sulfoxide (DMSO) and then growth medium, and added to cells. To measure proliferation of metabolically active cells, plates were incubated for 72 h prior to addition of Alamar Blue (Life Technologies). Plates were read after 4 additional hours of incubation at 37 °C using a Bio-Tek Synergy HT plate reader (Bio-Tek, Winooski, VT, USA). Data were analyzed and graphed using GraphPad Prism Software (GraphPad Software, La Jolla, CA, USA).

### 4.4. Cell Survival Assay

Cells were seeded in 96-well plates and treated with DMSO, BiQ-1, BiQ-2 and MonoQ as described above. Cells were incubated for 72 h, and then counted using trypan blue exclusion to identify blue dead cells on the Countess automated cell counter (Life Technologies). Cell counts were performed in duplicate, and the averages were graphed using GraphPad Prism software.

### 4.5. Clonogenic Assays

MOLM-14 and THP-1 cells were treated with either DMSO control or BiQ-1 at concentrations of 0.1, 2, and 5 μM for 24 h. The cells were then washed and resuspended in growth medium and methylcellulose with or without DMSO control or BiQ-1 treatments. After 7 days for MOLM-14 and 12 days for THP-1, the experiments were terminated with addition of 1.0 mg/mL iodonitrotetrazolium chloride [29] (INT, Sigma Aldrich, St. Louis, MO, USA) and read 24 h later with an automated colony counter (Synbiosis, Frederick, MD, USA).

### 4.6. Western Blotting

The effect of BiQ-1 on apoptosis-related and reactive oxygen species (ROS)-related protein expression was tested in AML cell lines and primary cells [23]. Total protein extracts were prepared using radioimmunoprecipitation assay (RIPA) buffer (Sigma Aldrich) supplemented with Complete Mini™ protease inhibitor and PHOStop™ phosphatase inhibitors (Roche). Equal amounts of protein (up to 25 µg) were separated on 4%–12% NuPAGE gels in 1 × 3-(*N*-morpholino) propanesulfonic acid (MOPS) or 1 × 2-(*N*-morpholino) ethanesulfonic acid (MES) buffer (Invitrogen, Waltham, MA, USA) and transferred onto polyvinyl difluoride (PVDF) membranes (Millipore). Membranes were blocked with 5% dry milk in 1 × tris-buffered saline (TBS)/0.1% Tween 20 (TBST) for at least one hour at room temperature (rt) and incubated with human specific primary antibodies to caspase-3 (Cell Signaling Technologies, Danvers, MA, USA), Mcl-1 (Santa Cruz Biotechnology, Dallas, TX, USA), nuclear factor (erythroid-derived 2)-like 2 (Nrf2, H-300) (Santa Cruz), Kelch like-ECH-associated protein 1 (Keap1, H-300) (Santa Cruz), NQO1 (Abcam, Cambridge, MA, USA) or mouse anti-β-actin (Sigma Aldrich) overnight at 4 °C. Membranes were washed three times in TBST and incubated with a horseradish peroxidase-conjugated secondary anti-rabbit or anti-mouse antibody (Cell Signaling Technologies) for 1 h at rt. Blots were washed again and the signal was detected with SuperSignal West Femto Chemiluminescent Substrate (Pierce Biotechnology, Rockford, IL, USA) and exposed to HyBlot CL^®^ autoradiography film (Denville Scientific, Holliston, MA, USA).

### 4.7. Flow Cytometric Analysis of Apoptosis and Mitochondrial Membrane Potential

AML cell lines and primary cells were treated with DMSO control or 5, 10, or 20 μM BiQ-1. At 6, 24 and 48 h, cells were harvested, washed and stained using the Annexin V-FITC apoptosis detection Kit I (BD Biosciences, San Jose, CA, USA) and the Flowcollect™ Mitopotential Red Kit (Millipore, Billerica, MA, USA) according to the manufacturers’ recommendations. Samples stained for Annexin V-FITC analysis were acquired on a FACscan (BD Biosciences) and samples stained for mitochondrial membrane potential analysis were acquired on an LSR II (BD Biosciences) [7]. Acquired samples were analyzed using FlowJo software (Tree Star, Ashland, OR, USA).

### 4.8. Measurement of Cellular Reactive Oxygen Species (ROS)

THP-1 and MOLM-14 cells in logarithmic growth phase were centrifuged and re-suspended in PBS at one million cells per milliliter (mL). Cells were preloaded with 2′,7′-dichlorodihydrofluorescein diacetate (H_2_DCFA, Life Technologies) at a final concentration of 2 μM and incubated at 37 °C in the dark for 25 min. Cells were centrifuged and resuspended at one million per ml in phenol red-free RPMI with 10% FBS and again incubated at 37 °C in the dark for 25 min. Treatments were prepared at 2 × concentration in phenol red-free RPMI and added to each well to yield 1 × concentrations. Cells were incubated in the dark at 37 °C and measurement of ROS was carried out on a FACSCanto (BD Biosciences) at serial time points post treatment.

### 4.9. Uptake of Fluorescent ATP-Binding Cassette (ABC) Protein Substrates

To measure the effect of BiQ-1 on uptake of fluorescent ABC protein substrates, K562 cells transfected with ABCB1 (K562/ABCB1) or ABCG2 (K562/ABCG2) (1 × 10^6^) were incubated for 30 min at 37 °C with BiQ-1 (50 µM) in the presence or absence of the ABCB1-specific transport inhibitor PSC-833 or the ABCG2-specific transport inhibitor fumetrimorgin C, respectively, as previously described [30]. Subsequently, cells were washed twice and resuspended in PBS and kept on ice until analysis. Cells were analyzed on a FACSCanto II flow cytometer and analyzed using FlowJo software. Substrate content after uptake with and without inhibitors was compared using the Kolmogorov-Smirnov statistic, expressed as a D-value ranging from 0 (no difference) to 1 (no overlap) [31], with D-values ≥ 0.2 indicating significant modulation, as previously described [32].

### 4.10. In Vivo Tolerability Studies

The *in vivo* tolerability of BiQ-1 was assessed by intraperitoneal (IP) administration into six-week-old female NIH Swiss mice. BiQ-1 was formulated in 10% DMSO, 10% cremophor and 80% saline and dosed as a 10 mL/kg dosing solution. The mice were dosed IP once daily for five days, followed by two days off, and subsequently dosed daily for three more days. During the study, mice were weighed daily and observed for changes in appearance and behavior. One hour after the last dose, mice were euthanized by CO_2_ inhalation and exsanguinated. Whole blood (~200 µL) was placed in Li-EDTA tubes (BD) and shipped to BioReliance (Rockville, MD, USA) for complete blood count (CBC) determination. Serum was isolated after clotting by spinning blood at 14,000 rpm for 8 min. Serum was snap frozen and stored at −20 °C and shipped to BioReliance for a clinical chemistry panel including renal and hepatic function tests.

### 4.11. Statistical Analysis

Stata Software version 13.0 (StataCorp, College Station, TX, USA) and Graphpad Prism Software were used for analyses. Mean IC_50_ values for cell lines and primary cells were compared with the values for normal bone marrow using one sample *t*-test. All *p*-values are two-sided, and a *p* < 0.05 is considered significant. For other biological assays, either two-tailed student *t*-test or ANOVA (Tukey post-test) analyses were used.

## References

[1] Buchner T., Schlenk R.F., Schaich M., Dohner K., Krahl R., Krauter J., Heil G., Krug U., Sauerland M.C., Heinecke A. (2012). Acute myeloid leukemia (AML): Different treatment strategies *versus* a common standard arm—Combined prospective analysis by the german aml intergroup. J. Clin. Oncol..

[2] Emadi A., Karp J.E. (2014). The state of the union on treatment of acute myeloid leukemia. Leuk. Lymphoma.

[3] Siegel R.L., Miller K.D., Jemal A. (2015). Cancer statistics, 2015. CA Cancer J. Clin..

[4] Holohan C., van Schaeybroeck S., Longley D.B., Johnston P.G. (2013). Cancer drug resistance: An evolving paradigm. Nat. Rev. Cancer.

[5] Sallmyr A., Fan J., Datta K., Kim K.T., Grosu D., Shapiro P., Small D., Rassool F. (2008). Internal tandem duplication of FLT3 (FLT3/ITD) induces increased ROS production, DNA damage, and misrepair: Implications for poor prognosis in AML. Blood.

[6] Fan J., Li L., Small D., Rassool F. (2010). Cells expressing FLT3/ITD mutations exhibit elevated repair errors generated through alternative nhej pathways: Implications for genomic instability and therapy. Blood.

[7] Emadi A., Sadowska M., Carter-Cooper B., Bhatnagar V., van der Merwe I., Levis M.J., Sausville E.A., Lapidus R.G. (2015). Perturbation of cellular oxidative state induced by dichloroacetate and arsenic trioxide for treatment of acute myeloid leukemia. Leuk. Res..

[8] Pei S., Minhajuddin M., Callahan K.P., Balys M., Ashton J.M., Neering S.J., Lagadinou E.D., Corbett C., Ye H., Liesveld J.L. (2013). Targeting aberrant glutathione metabolism to eradicate human acute myelogenous leukemia cells. J. Biol. Chem..

[9] Sriskanthadevan S., Jeyaraju D.V., Chung T.E., Prabha S., Xu W., Skrtic M., Jhas B., Hurren R., Gronda M., Wang X. (2015). Aml cells have low spare reserve capacity in their respiratory chain that renders them susceptible to oxidative metabolic stress. Blood.

[10] Powis G. (1989). Free radical formation by antitumor quinones. Free Radic. Biol. Med..

[11] Driscoll J.S. (1974). Quinone structure-antitumor activity relationships. Cancer Chemother. Rep. Part 2.

[12] Keinan S., Paquette W.D., Skoko J.J., Beratan D.N., Yang W., Shinde S., Johnston P.A., Lazo J.S., Wipf P. (2008). Computational design, synthesis and biological evaluation of para-quinone-based inhibitors for redox regulation of the dual-specificity phosphatase Cdc25B. Org. Biomol. Chem..

[13] Wellington K.W. (2015). Understanding cancer and the anticancer activities of naphthoquinones—A review. R. Soc. Chem. (RSC) Adv..

[14] Decosterd L.A., Parsons I.C., Gustafson K.R., CardellinaII J.H., McMahon J.B., Cragg G.M., Murata Y., Pannell L.K., Steiner J.R., Clardy J. (1993). Structure, absolute stereochemistry, and synthesis of conocurvone, a potent, novel HIV-inhibitory naphthoquinone trimer from a *Conospermum* sp.. J. Am. Chem. Soc..

[15] Dai J.R., Decosterd L.A., Gustafson K.R., Cardellina J.H., Gray G.N., Boyd M.R. (1994). Novel naphthoquinones from *Conospermum incurvum*. J. Nat. Prod..

[16] Emadi A., Harwood J.S., Kohanim S., Stagliano K.W. (2002). Regiocontrolled synthesis of the trimeric quinone framework of conocurvone. Org. Lett..

[17] Stagliano K.W., Lu Z., Emadi A., Harwood J.S., Harwood C.A. (2004). Effect of methoxyl group position on the regioselectivity of ammonia substitution reactions involving 3,3′-dichloro-2,2′-binaphthoquinones. J. Org. Chem..

[18] Stagliano K.W., Emadi A., Lu Z., Malinakova H.C., Twenter B., Yu M., Holland L.E., Rom A.M., Harwood J.S., Amin R. (2006). Regiocontrolled synthesis and HIV inhibitory activity of unsymmetrical binaphthoquinone and trimeric naphthoquinone derivatives of conocurvone. Bioorg. Med. Chem..

[19] Ross A.E., Emadi A., Marchionni L., Hurley P.J., Simons B.W., Schaeffer E.M., Vuica-Ross M. (2011). Dimeric naphthoquinones, a novel class of compounds with prostate cancer cytotoxicity. BJU Int..

[20] Emadi A., le A., Harwood C.J., Stagliano K.W., Kamangar F., Ross A.E., Cooper C.R., Dang C.V., Karp J.E., Vuica-Ross M. (2011). Metabolic and electrochemical mechanisms of dimeric naphthoquinones cytotoxicity in breast cancer cells. Bioorg. Med. Chem..

[21] Emadi A., Ross A.E., Cowan K.M., Fortenberry Y.M., Vuica-Ross M. (2010). A chemical genetic screen for modulators of asymmetrical 2,2′-dimeric naphthoquinones cytotoxicity in yeast. PLoS ONE.

[22] Bey E.A., Reinicke K.E., Srougi M.C., Varnes M., Anderson V.E., Pink J.J., Li L.S., Patel M., Cao L., Moore Z. (2013). Catalase abrogates β-lapachone-induced PARP1 hyperactivation-directed programmed necrosis in NQO1-positive breast cancers. Mol. Cancer Ther..

[23] Niture S.K., Khatri R., Jaiswal A.K. (2014). Regulation of Nrf2—An update. Free Radic. Biol. Med..

[24] Fletcher J.I., Haber M., Henderson M.J., Norris M.D. (2010). ABC transporters in cancer: More than just drug efflux pumps. Nat. Rev. Cancer.

[25] Elangovan S., Hsieh T.C. (2008). Control of cellular redox status and upregulation of quinone reductase NQO1 via Nrf2 activation by α-lipoic acid in human leukemia HL-60 cells. Int. J. Oncol..

[26] Irwin M.E., Rivera-Del Valle N., Chandra J. (2013). Redox control of leukemia: From molecular mechanisms to therapeutic opportunities. Antioxid. Redox Signal..

[27] Duthie S.J., Grant M.H. (1989). The role of reductive and oxidative metabolism in the toxicity of mitoxantrone, adriamycin and menadione in human liver derived Hep G2 hepatoma cells. Br. J. Cancer.

[28] Anusevicius Z., Nivinskas H., Sarlauskas J., Sari M.A., Boucher J.L., Cenas N. (2013). Single-electron reduction of quinone and nitroaromatic xenobiotics by recombinant rat neuronal nitric oxide synthase. Acta Biochim. Pol..

[29] Meyer M., Rubsamen D., Slany R., Illmer T., Stabla K., Roth P., Stiewe T., Eilers M., Neubauer A. (2009). Oncogenic ras enables DNA damage- and p53-dependent differentiation of acute myeloid leukemia cells in response to chemotherapy. PLoS ONE.

[30] Sen R., Natarajan K., Bhullar J., Shukla S., Fang H.B., Cai L., Chen Z.S., Ambudkar S.V., Baer M.R. (2012). The novel BCR-ABL and FLT3 inhibitor ponatinib is a potent inhibitor of the MDR-associated ATP-binding cassette transporter ABCG2. Mol. Cancer Ther..

[31] Young I.T. (1977). Proof without prejudice: Use of the Kolmogorov-Smirnov test for the analysis of histograms from flow systems and other sources. J. Histochem. Cytochem..

[32] Minderman H., Suvannasankha A., O’Loughlin K.L., Scheffer G.L., Scheper R.J., Robey R.W., Baer M.R. (2002). Flow cytometric analysis of breast cancer resistance protein expression and function. Cytometry.

